# Metabolomic Markers for Predicting Preeclampsia in the First Trimester of Pregnancy: A Retrospective Study

**DOI:** 10.3390/molecules27082475

**Published:** 2022-04-12

**Authors:** Ekaterina V. Ilgisonis, Raisa Shalina, Nigyar Kasum-Zade, Kristina G. Burkova, Oxana P. Trifonova, Dmitry L. Maslov, Anna L. Kaysheva, Sergey S. Markin

**Affiliations:** 1 V.N. Orekhovich Institute of Biomedical Chemistry, 119121 Moscow, Russia; ilgisonis.ev@gmail.com (E.V.I.); oxana.trifonova@gmail.com (O.P.T.); dlmaslov@mail.ru (D.L.M.); comphos@mail.ru (S.S.M.); 2 Department of Obstetrics and Gynecology, Pirogov Russian National Research Medical University, Ostrovitianov Str. 1, 117997 Moscow, Russia; raisa.shalina@gmail.com (R.S.); nk260590@gmail.com (N.K.-Z.); burkovakristina@mail.ru (K.G.B.)

**Keywords:** preeclampsia, metabolites, digital medicine, direct mass spectrometry

## Abstract

We sought to identify the characteristic metabolite profile of blood plasma samples obtained from patients with preeclampsia. Direct high-resolution mass spectrometry was used to analyze samples from 79 pregnant women, 34 of whom had preeclampsia. We performed a comparative analysis of the metabolite profiles and found that they differed between pregnant women with and without preeclampsia. Lipids and sugars were identified as components of the metabolite profile that are likely to be associated with the development of preeclampsia. While PE was established only in the third trimester, a set of metabolites specific for the third trimester, including 2-(acetylamino)-1,5-anhydro-2-deoxy-4-O-b-D-galactopyranosyl-D-arabino-Hex-1-enitol, N-Acetyl-D-glucosaminyldiphosphodolichol, Cer(d18:0/20:0), and allolithocholic acid, was already traced in the first trimester. These components are also likely involved in lipid metabolism disorders and the development of oxidative stress.

## 1. Introduction

Preeclampsia (PE) is a hypertensive disorder that accompanies some pregnancies. In recent years, there has been no significant decrease in the incidence of PE [1]. PE complicates the course of 2–8% of pregnancies and is associated with serious complications, such as stroke, fatty hepatosis, and renal failure [2]. Currently, hypertensive complications during pregnancy, particularly PE, rank second in the world among the causes of maternal mortality. Every year, 70,000 pregnant women die from PE and its complications worldwide [3].

Despite a large number of studies, the etiology of this disease is not fully understood. Both clinical symptoms (arterial hypertension and severe edema) and laboratory symptoms are used to diagnose PE. The main laboratory symptoms of PE include hypercoagulation and cellular hemostasis, which can be indicated by various markers, including thrombocytopenia, proteinuria, increased creatinine, and hepatic enzymes. Methods characterizing the state of the fetus, such as ultrasound fetometry and Doppler ultrasound, are highly important for assessing the severity of PE. The severity is determined by assessing the extent to which certain parameters are altered.

In recent years, the prediction of PE in the first trimester of pregnancy has become possible as a result of to the study of genetic markers, placental and vascular proteins, and blood flow in the uterine vessels. In practical medicine, prognostic programs (Astraia) have been introduced and used during the first prenatal screening, capable of calculating the risks of developing early and late PE [4].

Serological markers of PE that meet the clinical requirements for selectivity, accuracy, and reliability are still absent in practice. In the PubMed database, using the search phrase “preeclampsia molecular diagnostics”, we identified more than 600 published scientific studies on the prediction and identification of promising candidate molecular markers for PE diagnosis. Most of the published studies investigated the molecular mechanisms underlying PE development and the prognostic markers of PE in the early stages of pregnancy. Although metabolic biomarkers for PE are not yet widely used in clinical practice, a literature analysis showed that metabolomics can potentially become a clinical tool for predicting and diagnosing PE and clarifying the etiology and pathogenesis of the disease [5,6]. 

Previously, Bahado-Singh et al. investigated the plasma metabolome of 60 pregnant women with PE using the 1H NMR (NOESY) method [7]. The study showed a significant increase in the levels of citrate and 3-hydroxyisovalerate (sensitivity, 75.9%; specificity, 95.1%; overall accuracy, 90.4%) in pregnant women with PE. In a similar study, Austdal et al. observed an increase in the content of very low density lipoproteins (VLDLs) and low-density lipoproteins (LDLs) (sensitivity, 90%; specificity, 100%; overall accuracy, 95%) in blood serum samples from 10 pregnant women with PE [8]. The same scientific group, using the HR-MAS MRS method [9], reported a significant increase in the levels of glycerophosphocholine, phosphocholine, and aspartate in the placenta of 19 pregnant women with PE. Odibo et al. analyzed 41 blood serum samples from pregnant women with PE via LC–MS/MS and demonstrated an increase in the levels of hydroxyhexanoylcarnitine, phenylalanine, glutamate, and alanine (sensitivity, 50%; specificity, 80%; overall accuracy, 82.3%) [10]. In summary, we conclude that lipids and associated metabolites are the most frequently detected compounds associated with the development of PE [5].

In a comparative Ross study of the metabolic composition of serum samples obtained from 129 women with a history of preterm birth, including 43 women diagnosed with preeclampsia, HPLC–MS/MS (Orbitrap) analysis revealed a PE-specific increase in 5-α-pregnan-3β,20 α-diol disulfate levels and a decrease in the levels of 1-linoleoylglycerophosphoethanolamine and octadecanedioate [11]. The authors proposed these metabolites as candidate markers for PE detection in pregnant women [11]. In another MS profiling of blood serum samples using a time-of-flight quadrupole detector in combination with HPLC (LC–Q-TOF MS), PC (14:0/00), proline betaine, and proline were identified as potential biomarkers for the diagnosis and prognosis of preeclampsia [12]. Notably, direct quantitative MS analysis using eight isotope-labeled standards of the metabolite composition from the blood samples of patients with PE revealed increased blood levels of hydroxyhexanoylcarnitine, alanine, phenylalanine, and glutamate [10]. 

In general, the authors of the above studies noted the significance of further studies on changes in the metabolic composition of the blood that accompanies the development of PE. The current literature makes it possible to identify metabolic pathway disorders that are likely to be significant in the development of PE, including insulin resistance, disorders of energy metabolism, oxidation processes, lipid metabolism, and activation of inflammatory processes [11,13]. 

There is an extremely urgent need to develop effective methods for identifying patients with a high risk of developing PE and for early molecular diagnosis. The study of the biological phenomena accompanying PE, primarily at the metabolic level, may lead to significant advances in the study of PE pathogenesis. The aim of this study was to identify predictors of PE based on a comparative mass spectrometric analysis of the metabolite composition of plasma samples from pregnant women with and without PE.

## 2. Results

### 2.1. Population Characteristics of the Study Participants

Analysis of the clinical and anthropometric characteristics of the patients (Table 1) showed that the average age in the groups, regardless of the outcome of pregnancy, did not differ significantly. Most of the patients were between the ages of 19 and 34 years. An age of 40 years or more was observed in five (16.1%) patients with PE.

Approximately 61.2–76.1% of preeclampsia cases, especially severe, may be attributed to an underlying extragenital pathology [14]. In this regard, extragenital pathology (such as arterial hypertension, chronic kidney disease, autoimmune diseases, diabetes mellitus, body mass index (BMI) of 35 kg/m^2^, age 40 years or older) is a risk factor for the development of the disease according to the National Institute for Health and Care Excellence, American College of Obstetricians and Gynecologists [15,16]. The analysis of extragenital pathologies revealed that the frequency of hereditary thrombophilia in a patient with PE was almost twice as that in healthy pregnant women (*p* < 0.05).

Changes in vital organs in the presence of an extragenital pathology are an additional factor in multiple organ failure. It is difficult to isolate patients with severe preeclampsia without an extragenital pathology.

The effects of hereditary changes in extragenital pathology and preeclampsia are of great interest. Group 4 included one patient with a BMI of 45.3 kg/m^2^, whereas the BMI varied from 27 to 35 kg/m^2^ in the rest of the groups.

Chronic arterial hypertension (CAH) and urinary system diseases were observed only in patients with PE. In 16% of the cases, previous pregnancies were complicated by PE of varying severity. In six (19%) patients with PE, pregnancy was achieved using assisted reproductive technologies. In the third trimester, in healthy pregnant women, fetal growth retardation (FGR) was detected in one patient (5%), and severe PE was observed in six patients (19%). It should be noted that in three patients examined in the first trimester who subsequently developed PE, there were no clinical or laboratory signs warranting their inclusion in the PE risk group, according to the Astraia program.

Severe preeclampsia develops in patients with chronic arterial hypertension and lipid metabolism disorder. In group 2, the pregnancy ended in extreme preterm labor, and the body weights of the newborns were 890 and 990 g, respectively. Seven patients delivered at 33–36 weeks of gestation, and the body weight of the children ranged from 1810 to 2570 g.

After delivery, the premature babies were transferred to the intensive care unit and then discharged. The rest of the patients delivered at 37–38 weeks of gestation.

Diseases of the urinary system occurred in only three patients in group 4. A history of pyelonephritis was noted, with at least one relapse in 2–3 years. The last relapse occurred 1–2 years before pregnancy.

This study did not include patients with glomerulonephritis, renal failure, or a history of nephrectomy.

### 2.2. Altered Plasma Metabolites in the Different Groups

Nine endogenous metabolites (lipids by nature and sugars) were presumably annotated, and their frequency varied in response to the presence or absence of PE (Table 2).

**Figure 1:**
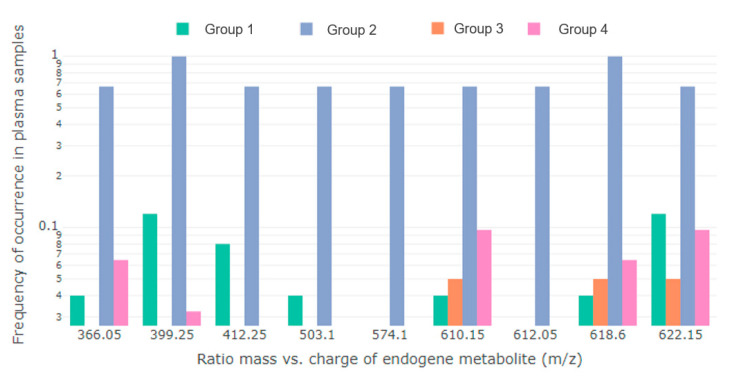
Frequencies (log scale) of nine *m/z* characteristics among four groups of patients without averaging over technical repetitions (TR). TR were considered as independent measurements. Group 1 and 2—a series of blood samples from patients in the control group and with developed PE, examined in the first trimester, respectively; Group 3 and 4—a series of blood samples from the study participants in the control group and with PE in the third trimester of pregnancy, respectively.

The metabolites (Table 1) were detected in comparison groups (Figure 1) with different frequencies.

Based on the findings, each of the proposed sets of endogenous metabolites (Table 1) was effective for assessing the risk of developing PE in the first trimester. There was no significant difference in the frequency of metabolites in the third trimester between healthy pregnant women and women with PE (*p* > 0.05).

The biological significance of the revealed set of metabolites in the pathogenesis of PE was analyzed (Table 1). This set is consistent with data reported in the literature. These metabolites are involved in biological processes that are likely to accompany the development of this disease.

## 3. Discussion

It is now known that during the normal course of pregnancy and during the development of PE, there are changes in the activity of the mother’s immune system and in the modulation of metabolic processes. Thus, the development of PE accompanies immune changes that contribute to the secretion of factors from CD4^+^ T-helper cells, cytotoxic NK cells, and autoreactive B cells that stimulate innate immune activation and inflammatory reactions in the maternal bloodstream and uteroplacental region. These immune changes lead to impaired trophoblast invasion into the spiral arteries and their remodeling during early pregnancy. Placental ischemia occurs due to decreased blood flow, which increases oxidative stress and stimulates the release of anti-angiogenic factors caused by hypoxia. This shift in the immune response during PE suggests that inflammation plays a key role in the development and progression of the disease [17].

Among the metabolites that exhibited differences between the comparison groups, long-chain fatty acids, phospholipids, and sphingolipids were detected, including neuroprotectin D1 (HMDB0003689), N-acetyl-D-glucosaminylphosphatidylinositol, and N-(eicaninosanoyl)-sphinganine (d18:0/20:0) (HMDB0011764), which are involved in stabilizing the structure of cell and organelle membranes. Normal pregnancy is associated with a wide range of metabolic adaptations in the mother’s body, including increased lipid and lipoprotein metabolism. In PE, an increase in the content of long-chain fatty acids and phospholipids can disrupt the structural and functional properties of placental membranes.

Carnitine derivatives, such as dodecanedioylcarnitine (HMDB0013327), were included in the set of endogenous metabolites. Carnitine plays an important role in the metabolism of fatty acids and is required for their transport between the inner and the outer mitochondrial membranes. Carnitine also binds toxic metabolites, including acyl groups, which are subsequently eliminated in the form of acylcarnitines in urine [18]. Fatty acids play a vital role in pregnancy as a metabolic energy source for the placenta [19]. Fetal disorders of mitochondrial fatty acid oxidation have been associated with obstetric complications, including preeclampsia, hemolysis, placental floor infarction, and acute fatty liver of pregnancy [18,19]. Several studies suggested that an elevated acylcarnitine level is a potential biomarker of preeclampsia [18,20,21].

Keller and Thiele reported that the concentration of carnitine and its derivatives in the blood plasma of patients noticeably decreases during pregnancy [18,22]. With PE, there is a significant increase in the levels of carnitine and its derivatives, primarily acylcarnitines [18]. This increase, along with an increase in the total lipid level, confirms the role of lipid metabolism disorders in the pathophysiology of PE. It is assumed that toxic metabolites (derivatives of carnitine, etc.), resulting from the abnormal peroxidation of fatty acids in the placenta, cause endothelial dysfunction [18]. A tandem mass spectrometry (HPLC–MS/MS)-based study also substantiated this finding, except for the increase in plasma carnitine levels in the group of pregnant women with preeclampsia. At the same time, the content of free carnitine and short- and long-chain acylcarnitine increased by approximately 50% compared to that in the control group [18]. 

Of interest is the component of the estriol-16-glucuronide (HMDB0006766) set, which belongs to the steroid hormone group that determines adequate vascular adaptation and blood supply in the uterus and placenta. This endogenous metabolite is a component of steroid hormone biosynthesis (KEGG C05504). Estriol-16-glucuronide is a derivative of estriol. The glucuronidation reaction leads to the increased water solubility of the metabolite compared to the intact non-derivatized form and is a way to eliminate endogenous metabolites or xenobiotics by the kidneys. Estriol is one of the three estrogens and is produced by the placenta in significant amounts only during pregnancy. The relationship between steroid hormone levels and maternal cardiovascular and placental function warrants further study [23,24]. A recent longitudinal study substantiated the importance of detecting estriol-16-glucuronide along with tetrahydrodeoxycorticosterone and progesterone in the blood of pregnant women to predict the gestational age in a metabolic clock model [25]. The authors did not specify the biological role of estriol-16-glucuronide but pointed out its important role as a participant in hormonal metabolism. The role of estriol-16-glucuronide in maintaining placental perfusion has been known since 1932 [26]. Due to its vasodilating effect, the increase in estrogen levels during pregnancy helps maintain the function of the uteroplacental vessels [27,28]. 

Clinical studies have suggested that decreased estrogen levels impair placental perfusion during pregnancy [29,30,31]. As noted earlier, the molecular mechanisms leading to changes in estrogen levels remain unclear [32]. Changes in estrogen levels may be associated with changes in metabolism during pregnancy [33]. 

Bile acid (allolithocholic acid) (HMDB0000381) belongs to a set of endogenous metabolites. Some studies have reported an increase in the level of bile acid in the blood of pregnant women with PE [34,35]. Pathological mechanisms that cause abnormal liver function in PE may predispose patients to cholestasis [34]. In the absence of apparent clinical and biochemical signs, in some cases, there is an increase in the bile acid levels in the blood serum of women with preeclampsia. The authors suggested that liver failure in some women with preeclampsia may lead to the development of cholestasis [34]. Many studies have noted the importance of monitoring the bile acid levels in the blood of pregnant women to identify possible maternal liver dysfunction [36,37]. 

Another component of the metabolite list, biotinyl-5′-AMP (HMDB0004220), is the active form of biotin in humans. In cells, biotin is necessary for maintaining metabolic homeostasis and plays an important role in gluconeogenesis, fatty acid synthesis, and carbohydrate metabolism, acting as a cofactor for five carboxylases: pyruvate carboxylase, propionyl CoA carboxylase, methyl crotonyl CoA carboxylase, and two forms of acetyl CoA carboxylases (KEGG C05921) [38]. The literature discusses the importance of impaired carbohydrate metabolism (glycolysis/gluconeogenesis) and the content of adenine nucleotides, including ADP-glucose, for the energy state of the placenta in PE. To date, there is no direct evidence of a relationship between the concentration of adenine nucleotides and energy balance (an indicator of the redox state of cytoplasmic reduced nicotinamide adenine dinucleotide/nicotinamide adenine dinucleotide). Metabolic abnormalities in carbohydrate metabolism are likely to develop in the placenta of mothers with PE, which can subsequently lead to changes in the energy state of the placenta [39,40].

Thus, the experimental data obtained in this study are consistent with the current understanding of the molecular mechanisms of the development of preeclampsia. We pay particular attention to the prevalent change in lipid metabolism as closely related to the metabolism of long-chain fatty acids and steroid hormones, as well as to carbohydrate metabolism, especially, gluconeogenesis.

Understanding the pathological events at the molecular and cellular level will allow ushering new approaches for the determination of PE risk groups during gestation. The results obtained are of interest for the development of new approaches for the detection of molecular factors facilitating the development of the disease at an early stage.

## 4. Materials and Methods

### 4.1. Demographics

The work was conducted at the GBUZ Center for Family Planning and Reproduction in Moscow and at the V.N. Orekhovich Institute of Biomedical Chemistry. A comparative analysis of the composition of endogenous metabolites in blood plasma samples was performed (Table 2). The study included 79 pregnant women in the first and third trimesters. The patients were divided into four groups, depending on the presence or absence of PE and the gestational age:

- Group 1 comprised 25 patients examined in the first trimester whose pregnancy proceeded without complications. Healthy children were born, with an average body weight of 3100 ± 200.0 g. The Apgar score was 8/9.
- Group 2 comprised 3 patients examined in the first trimester who developed moderate late PE in the third trimester. Delivery occurred at 37–39 weeks. The average body weight of the newborns was 3100 ± 220.0 g. The Apgar score was 8/9.
- Group 3 comprised 20 patients examined in the third trimester with uncomplicated pregnancies. Delivery occurred at 37–40 weeks. The average body weight of the newborns was 3500 ± 366.3 g. The Apgar score was 8/9.
- Group 4 included 31 patients admitted to the clinic during the third trimester (27–40 weeks). Ten (32.2%) patients had moderate preeclampsia, and 21 (67.8%) had severe preeclampsia. Patients with moderate preeclampsia delivered at 37–38 weeks. The body weight of the newborns was 2900–3000 g, with an Apgar score of 7/9 points. Patients with severe preeclampsia delivered at 27–36 weeks. Two births were exceptionally early (27–29 weeks, body weight, 890 g and 990 g), and 19 births occurred at 33–36 weeks (body weight, 1810–2600 g). Eleven premature infants with respiratory failure were observed and treated in the intensive care unit. All patients were discharged in satisfactory condition.

### 4.2. Sample Collection

Blood was collected in pre-chilled tubes with ethylenediaminetetraacetic acid (EDTA, Merck KGaA, Darmstadt, Germany), quickly mixed and centrifuged at 4 °C and 1500 rpm for 10 min; after centrifugation, the blood plasma samples were immediately collected and frozen. 

This study was approved by the independent Local Research Ethics Committee of the Pirogov Russian National Research Medical University (Protocol no. 169 issued on 20 November 2017, Moscow, Russia). Written informed consent was obtained from the patients and from healthy volunteers authorizing their participation in the study and the use of the biological material. All samples were deactivated prior to their use in the study to provide biological safety.

### 4.3. Sample Preparation for MS Analysis

To remove proteins and extract metabolites, 10 µL of each blood plasma sample was mixed with 10 µL of water (LiChrosolv; Merck KGaA, Darmstadt, Germany) and 80 µL of methanol (Fluka, Munich, Germany) and incubated at room temperature for 10 min. After incubation, the samples were centrifuged at 13,000× *g* (Centrifuge 5804R; Eppendorf AG, Hamburg, Germany) for 15 min at room temperature, and the resultant supernatants were then transferred to clean Eppendorf tubes. Before mass spectrometry analysis, each sample was diluted 50-fold with methanol containing 0.1% formic acid (Sigma-Aldrich, St. Louis, MO, USA), and the internal standard (IS) Losartan (C22H23ClN6O, *m/z* = 423.169, Merck KGaA, Darmstadt) was added to an end concentration of 10 ng/mL to obtain the solution to be analyzed. All chemicals and solvents were of HPLC and UHPLC grade.

### 4.4. Mass Spectrometry-Based Metabolite Registration

Mass spectrometry analysis of the samples was carried out by a hybrid quadrupole time-of-flight mass spectrometer (maXis Impact, Bruker Daltonics, Billerica, MA, USA) equipped with an electrospray ionization (ESI) source. The mass spectrometer was set up to detect ions with the mass-to-charge ratio (m/z) in the range from 50 to 1000 Da and mass accuracy up to 3 parts per million (ppm). The appropriate mass range of the mass spectrometer was previously calibrated by using ES Tuning Mix (Agilent). The spectra were acquired in the positive ion mode detection. The samples were injected into the ESI source using a glass syringe (Hamilton Bonaduz AG, Bonaduz, Switzerland) and a syringe injection pump (KD Scientific, Holliston, MA, USA) with a flow rate of 180 µL/h for 1 min. All samples were analyzed in random order and in two technical replicates.

### 4.5. Data Analysis and Metabolite Identification

Mass spectra were processed using DataAnalysis 4.1 software (Bruker Daltonics) to summarize the recorded signals. Additional internal calibration of the spectra was performed using characteristic high-intensity peaks over the entire range of the recorded *m/z* values. Peaks were detected using the following parameters: number of points per peak, 2; signal-to-noise ratio, 1; cutoffs for relative and absolute intensities, 0.01% and 100%, respectively. The intensities of the mass spectrometric peaks were normalized.

Estimation of the total number of ions of low-molecular-weight substances in the spectra corresponding to each sample. 

The number of ions registered in each sample allows the indirect identification of errors that occur during the collection, storage, and preliminary preparation of the sample and/or the correctness of the device settings selected for the measurements. A total of 14,087 ions were recorded. Of these, 30% were identified in all samples. For each sample, duplicate lists of the *m/z* value and intensity of the ions were generated to assess the reproducibility of the measurements. An ion was considered detected if its *m/z* value was registered in both technical replicates.

The list of masses was processed, and the chemical compounds were putatively identified using the resource http://www.mycompoundid.org (accessed on 10 December 2021). The list of detected *m/z* values was loaded into the search window, and the measurement accuracy was set to 2 ppm. Subsequently, the list of putatively identified metabolites was loaded automatically.

A total of 1234 metabolites were putatively identified. The frequency of the metabolites in each group was calculated. For the final analysis, we selected the metabolites that were absent from the “healthy” group but present in the PE group.

## 5. Conclusions

PE is a systemic disease and a leading cause of maternal and perinatal morbidity and mortality. The molecular basis for the development of this disease is unclear. Metabolic features during pregnancy complicated by PE include a decrease in cellular glucose uptake (glycolysis) and hyperlipidemia. In pregnant women with PE, an increase in the levels of circulating triglycerides and non-esterified free fatty acids is observed. The pathogenic process of PE begins in the first trimester of pregnancy. Therefore, the search for early molecular markers of PE is an important practical task. Blood plasma is an attractive source of candidate molecular markers and specific pathologies because it potentially contains molecules secreted by cells from diseased tissue. The study of the metabolite profile in women with PE will shed light on changes in tissue metabolism and helped identify critical changes associated with the body’s response to PE development.

We putatively identified nine endogenous metabolites in the blood samples of pregnant women in the first trimester who subsequently developed PE in the early stages of gestation. The frequency of occurrence of these metabolites differed between healthy patients and those who developed PE in the third trimester. Dodecanedioylcarnitine is probably involved in the disruption of the normal oxidation of fatty acids in the placenta; neuroprotectin D1, N-acetyl-D-glucosaminylphosphatidylinositol, and N-(eicosanoyl)-sphinganine cause endothelial dysfunction, impaired permeability, and impaired transport activity in the cell membrane; and biotinyl-5′-AMP and ADP-glucose participate in the metabolism of carbohydrates and modulate the energy balance in the placenta. The endogenous metabolites presented in this study explain the currently known pathophysiological processes associated with the disease and may be used to predict PE. Subsequent validation of the proposed combination of these easily analyzed metabolites is important.

## Figures and Tables

**Table 1:** Demography of the patients.

Characteristics		I Trimester		III Trimester	
		Group 1 without PE (n = 25)	Group 2 PE in 3rd Trimester (n = 3)	Group 3 without PE (n = 20)	Group 4 PE in 3rd Trimester (n = 31)
Age, years		31.6 (5,6)	29.0 (5,6)	32.7 (4,8)	32.6 (6)
PE in previous pregnancies		0	0	0	5 (16%)
Assisted reproductive technologies		2 (8%)	0	0	6 (19%)
BMI, kg/m^2^		21.9 (2,2)	22.8 (4,9)	21.3 (2,1)	28.4 (6,5)
Extragenital pathology	Diseases of the thyroid gland	0	0	2 (10%)	4 (13%)
	Hereditary thrombophilia	2 (8%)	0	2 (10%)	7 (23%)
	Chronic arterial hypertension	0	0	0	9 (29%)
	Diseases of the urinary system	0	0	0	3 (10%)
Parity	Primary pregnant	10 (40%)	2 (67%)	6 (30%)	16 (52%)

n (%), mean (SD).

**Table 2:** Frequency of endogenous metabolites detected in blood plasma samples during normal pregnancy and complicated PE.

Metabolite ID	Metabolite Name	Class	KEGG Compound ID	Nominal Mass *	Detected Mass **	I Trimester				III Trimester			
						Group 1		Group 2		Group 3		Group 4	
						Frequency	Number of Samples	Frequency	Number of Samples	Frequency	Number of Samples	Frequency	Number of Samples
HMDB0002282	2-(acetylamino)-1,5-anhydro-2-deoxy-4-O-b-D-galactopyranosyl-D-arabino-Hex-1-enitol	O-glycosyl compounds	N/A *	365.13	366.05	0.04	1	0.67	2	-	-	0.06	2
HMDB0003689	Neuroprotectin D1	Very long-chain fatty acids	N/A	360.23	399.25	0.12	3	1	3	-	-	0.03	1
HMDB0013327	Dodecanedioylcarnitine	Tricarboxylic acids	N/A	373.25	412.25	0.08	2	0.67	2	-	-	-	-
HMDB0006766	Estriol-16-Glucuronide	Steroids and steroid derivatives	C05504 Steroid hormone biosynthesis	464.20	503.1	0.04	1	0.67	2	-	-	-	-
HMDB0004220	Biotinyl-5′-AMP	Carboxylic acids and derivatives	C05921 Biotin metabolism	573.14	574.1	-	-	0.67	2	-	-	-	-
HMDB0001445	N-Acetyl-D-glucosaminyldiphosphodolichol	Prenol lipids	C04500 N-Glycan biosynthesis	587.23	610.15	0.04	1	0.67	2	0.05	1	0.1	3
HMDB0006557	ADP-glucose	Purine nucleotide sugars	C00498 ADP-glucose metabolism	589.08	612.05	-	-	0.67	2	-	-	-	-
HMDB0011764	Cer(d18:0/20:0)	Ceramides are also known as sphingolipids	N/A	595.59	618.6	0.04	1	1	3	0.05	1	0.06	2
HMDB0000381	Allolithocholic acid	Bile acid present in normal serum	N/A	376.30	622.15	0.12	3	0.67	2	0.05	1	0.1	3

Nominal mass *—theoretically predicted mass of the metabolite; Detected mass **—experimentally obtained mass of the metabolite. Differences between Nominal mass and Detected mass are due to the detection of Na + and K + adducts.

## Sample Availability

Plasma samples are available upon request from the authors.
